# Supplementary material for: Nanoscale origins of the damage tolerance of the high-entropy alloy CrMnFeCoNi
Source: Nat Commun. 2015 Dec 9;6:10143. doi: 10.1038/ncomms10143 (PMC4682111; doi:10.1038/ncomms10143)
Supplement: Supplementary Information — Supplementary Figures 1-3, Supplementary Note 1-3 and Supplementary References [file ncomms10143-s1.pdf]

Supplementary Figures

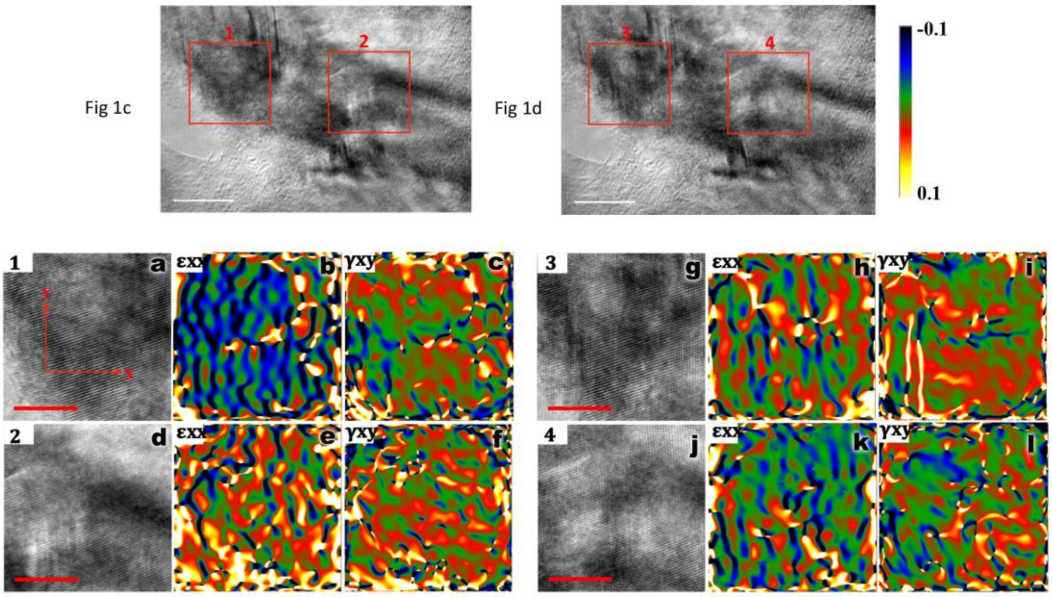

Supplementary Figure 1. Geometric phase analysis of images. Geometric phase analysis (GPA) on the HRTEM images extracted from the *in situ* videos (Supplementary Movie 1), which constitute Figs. 1c,d in the main paper. The areas, marked as “1” and “2”, are ~20 nm away from the crack tip. **a,d.** HRTEM images of areas “1” and “2”. **b,c.** Local lattice strain maps of normal strain along *x*-axis (<111> direction) and shear strain in area “1” in **c**. In the strain maps, colors for positive values represent tensile strain and those for negative values represent compressive strain. **e**,f. Srain maps of area 2 in **c**. **g,j.** HRTEM images taken from area “1” and “2” in Fig. 1d. **h,i,k,l**.) Strain maps of normal strain and shear strain of **g** and **j**, respectively. The scale bars in **c,d** represent 10 nm; the scale bars in **a, d, g, j** represent 5 nm.

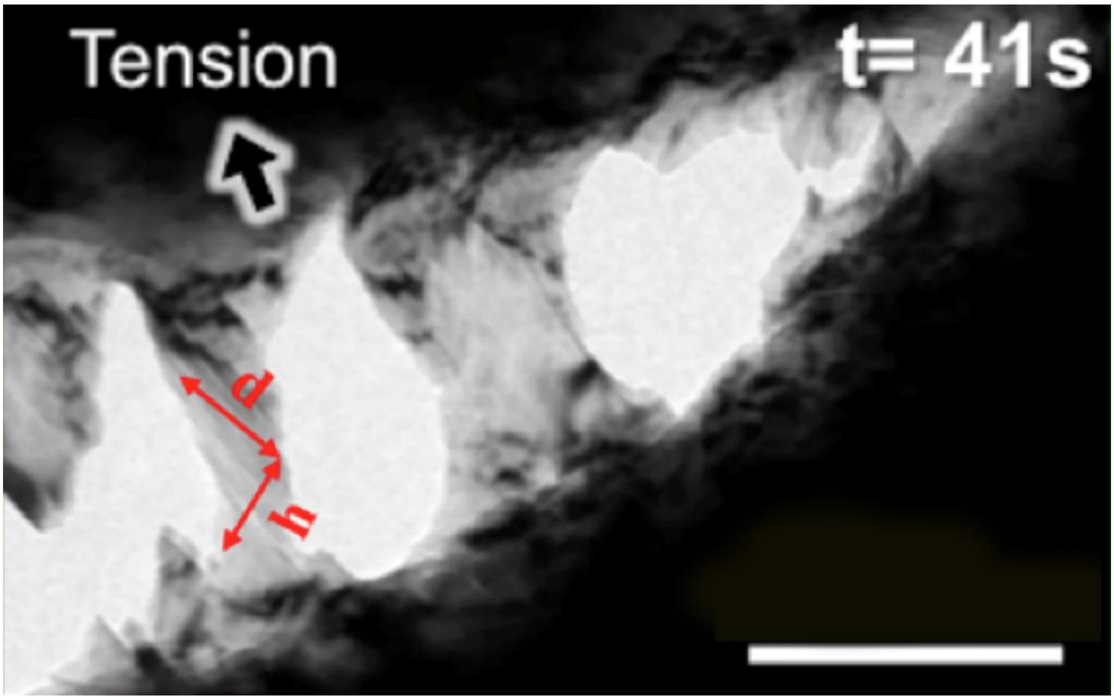

Supplementary Figure 2. Nano-bridging in the crack-tip region. *In situ* TEM image of nano-bridge ligaments deforming at the crack tip by twinning under load (scale bar represents 200 nm). For the purposes of the calculation of the energy dissipation by twinning vs. dislocation slip (see text), *d* and *l* can be considered to be the dimensions of the sample in which the dislocation glides (see Supplementary Notes 2).

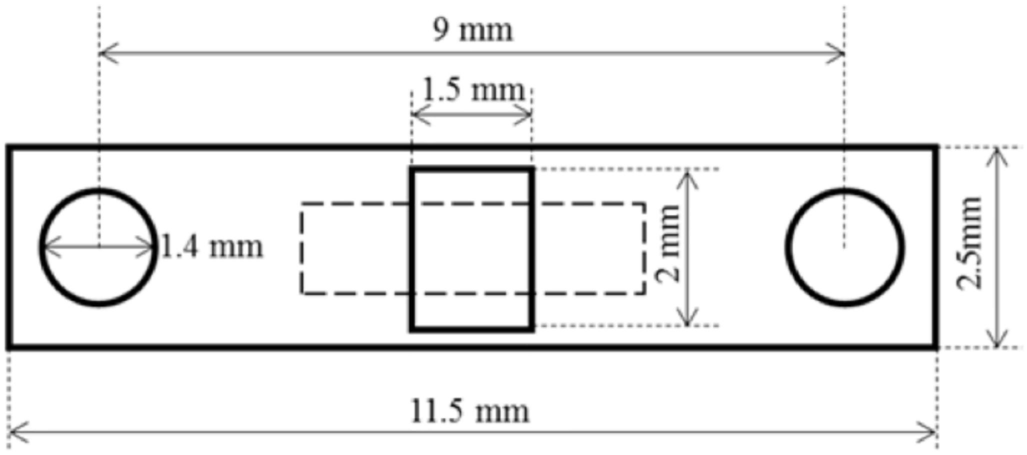

Supplementary Figure 3. Straining stage for in situ TEM straining tests. Schematic diagram (not to scale) of the stainless steel substrate used in the TEM straining stage for the *in situ* tensile experiments. The dashed rectangle indicates the approximate position of the HEA foil that was glued on the substrate across the rectangular window in the center that allowed for electron beam transmission. The loading pins of the *in situ* straining stage were inserted in the two circular holes shown at the ends of the substrate.

Supplementary Note 1. Strain analysis of the crack-tip region

Local lattice strain distribution maps were obtained from time-resolved HRTEM images (shown in Fig. 1c,d in the main text of the paper) using geometric phase analysis (GPA), as shown in Supplementary Figure 1. The *x*-axis is along the normal direction of the {111} plane on which Shockley partials glide during the opening of the crack. With the propagation of the crack tip, the strain (and therefore stress) states were clearly discernible with the different colors. Before the nucleation of Shockley partials, the local normal stresses along the <111> direction in region 1 are the most compressive (Supplementary Fig. 1b). It seems reasonable to assume that the area of enhanced stress concentration (the blue area) is where partial dislocations nucleated. After the nucleation of partials, the local lattice normal distortion became less severe (Supplementary Fig. 1h). The pronounced variation in shear strain γ_{xy} also indicates the nucleation of partials (Supplementary Fig. 1c,i). In region "2", which is $\sim$20 nm away from the crack tip, the strain state is complex due to the high density of dislocations (Supplementary Fig. 1e,f,k,l), in particular in the vicinity of the undissociated dislocations (Supplementary Fig. 1k).

Supplementary Note 2. Calculation of the energy dissipation achievable by twin and slip

The effectiveness of twinning in the nano-bridging ligaments can be estimated by the following considerations. The Taylor factor (M) links the single crystal shear strain (γ) to the polycrystalline tensile strain (ε) as follows: $\gamma = M \cdot \varepsilon$. Considering the fcc twinning shear of $\gamma = 1/\sqrt{2}$, and assuming an average Taylor factor of M = 3.06 for a randomly textured material, twinning can account for a maximum tensile strain of 0.23 ($\varepsilon = \gamma/M$), provided that the twinned volume is 100% of the total material; in reality, the strain that can be accommodated by deformation twinning will likely be well below this value.[1] In the current experiment where nano-twinned bridges are formed in the wake of the crack tip (Supplementary Fig. 2), if a twin of thickness 10 nm is formed in a bridging ligament, the volume fraction of the nano-twin will be ~10%, so the shear strain produced by the twin will be on the order of $\varepsilon \approx 2.3 \times 10^{-2}$.

Correspondingly, the shear strain for twins that are 5 and 2 nm thick will be ~1.15×10^{-2} and 0.46×10^{-2}, respectively.

By comparison, the shear strain produced by the motion of a full dislocation is $\gamma = b\Delta x/hd$, where b is the Burgers vector, Δx is the displacement of the dislocation, h and d are the parameters of the crystal in which the dislocation glides (as shown by the dimensions of the nano-bridge in Supplementary Fig. 2). Considering maximum values for the nano-bridge, $\Delta x_{max} = d$, such that γ_{max} ~ b/h ~ 0.004, one gets a macroscopic strain induced by the full dislocations of $\varepsilon = \gamma/M \approx 1.3 \times 10^{-3}$.

The Burgers vector of a partial dislocation is $^1/_6\, a[112]$, so the corresponding shear strain induced by a partial dislocation would be ~7.5×10^{-4}.

Another method to calculate the partial dislocation strain involves considering the plastic strains induced by the motion of a pre-existing partial which can be estimated as $\varepsilon = \rho_0 b_s d/M$, where ρ_0 is the initial dislocation density, b_s is the Burgers vector of Shockley partial dislocation, d is the grain size, and M is the Taylor factor).[1] For one partial dislocation gliding in the bridging ligament, $\rho_0 \approx 1/dh$, so $\varepsilon \approx b_s/hM = 7.5 \times 10^{-4}$.

In summary, the plastic strain produced by nano-twins in the crack-tip bridging ligaments is much larger than the strain induced by the motion of a full dislocation or partial dislocation through the nano-bridge. As the strain energy is a function of ε^2 and the elastic modulus, assuming that the modulus remains constant, the energy dissipation achieved by twinning would be much larger. Of course, the above comparison is confined to twinning and dislocation activity within the nano-bridges. Considerably more energy would be dissipated by dislocation activity in the plastic zone ahead of the crack tip than by nano-twinning within the fiber-like bridges.

Supplementary Note 3. Factors controlling the synergy of deformation mechanisms

As shown in the Supplementary Movie 1 and 2, there is a marked activation of

fast-moving partial dislocations at the early stage of deformation. However, since the full dislocations move slowly, forming localized bands of planar slip, the fast motion of partial dislocations is blocked at higher strains. In relatively simple terms, these observations may be rationalized as follows.

Whether a dislocation splits into two dislocations with smaller Burgers vectors depends on whether the resulting difference in their self-energies (which scales with the shear modulus and the square of the Burgers vector) is greater than the energy cost of creating the stacking fault. If dissociation is favored, the main factors that determine the spacing between the partials are (i) the repulsive force between them, which varies directly with the shear modulus and inversely with the separation distance and therefore favors a large separation, and (ii) the stacking fault energy, which favors a small separation distance. While it is conceivable that the local elastic modulus may vary in a high-entropy alloy, the repulsion between the partials is a result of long-range elastic interactions, which should depend on the average elastic modulus in a relatively large region and should thus not be very sensitive to the local variations in chemical composition. On the other hand, the stacking fault energy is a more local property that may strongly change depending on local chemical composition. Although the distribution of elements appears to be random and disordered in high-entropy alloys,[3,4] it is conceivable that the local stacking fault energy may vary from point to point. Indeed, recent HAADF/MAADF observations of dislocation core structures in this alloy seem to support this notion.[5] These local variations in stacking fault energy could account for the observed coexistence of full and partial dislocations.

Additionally, since the Peierls stress varies exponentially as ($-w/b$), where w is the dislocation width and b is the Burgers vector, it would be higher for a full dislocation than for its shorter partials. In other words, local chemical fluctuations could result in local regions of undissociated and dissociated dislocations with different mobilities. Their subsequent synergistic interactions could then produce the sequence of events shown in the movies discussed above. Systematic theoretical calculations, if possible, would certainly be helpful to understand local differences in the stacking fault energy and the stress to move different dislocations, but because of the complex nature of high

entropy alloy systems including chemical disorder, current simulation techniques are considered to not be good enough to handle such complex systems.

Supplementary References

1. Otto, F., Dlouhý, A., Somsen, Ch., Bei, H., Eggeler, G. & George E. P. The influences of temperature and microstructure on the tensile properties of a CoCrFeMnNi high-entropy alloy. *Acta Materialia* **61**, 5743-5755 (2013).
2. Lu, L., Chen, X., Huang, X. & Lu, K. Revealing the maximum strength in nanotwinned copper. *Science* **323**, 607-610 (2009).
3. Laurent-Brocq, M., Akhatova, A., Perrière, L., Chebini, S., Sauvage, X., Leroy, E. & Champion, Y. Insights into the phase diagram of the CrMnFeCoNi high entropy alloy. *Acta Materialia* **88**, 355-365 (2015).
4. Schuh, B., Mendez-Martin, F., Völker, B., George, E.P., Clemens, H., Pippan, R. & Hohenwarter, A. Mechanical properties, microstructures and thermal stability of a nanocrystalline CoCrFeMnNi high-entropy alloy after severe plastic deformation. *Acta Materialia* **96**, 258-268 (2015).
5. Smith, T. M., Esser, B. D., George, E. P., Otto, F., Ghazisaeidi, M., McComb, D. W. & Mills, M.J. HAADF/MAADF observations and image simulations of dislocation core structures in a high entropy alloy. *Microscopy & Microanalysis* **21** (Suppl 3), 2205-2206 (2015).
